# Astragalus polysaccharide enhances the therapeutic efficacy of cisplatin in triple-negative breast cancer through multiple mechanisms

**DOI:** 10.32604/or.2024.050057

**Published:** 2025-02-28

**Authors:** LI SUN, SHICHAO ZHUO, XIAOXIN LI, HUSHENG KONG, WEIWEI DU, CHONG ZHOU, JUNXING HUANG

**Affiliations:** 1 Department of Oncology, Taizhou People’s Hospital Affiliated to Nanjing University of Chinese Medicine, Taizhou, 225300, China; 2 Department of Pathology, Xuzhou Central Hospital Affiliated to Nanjing University of Chinese Medicine, Xuzhou, 221009, China; 3 Department of Oncology, Xuzhou Central Hospital Affiliated to Nanjing University of Chinese Medicine, Xuzhou, 221009, China; 4 Department of Radiotherapy, Xuzhou Central Hospital Affiliated to Nanjing University of Chinese Medicine, Xuzhou, 221009, China

**Keywords:** Triple-negative breast cancer (TNBC), Cisplatin (DDP), Astragalus polysaccharide (APS), T cell tumor infiltration, Combination treatment

## Abstract

**Background:**

Cisplatin (DDP) has been used in the treatment of various human cancers. However, DDP alone lacks efficacy in treating triple-negative breast cancer (TNBC), and its clinical application is often hampered by side effects. *Astragalus polysaccharide* (APS) is one of the active components extracted from *Astragalus membranaceus* and has gained attention for its various biological properties. This research is aimed to evaluate the effectiveness of a combination of APS and DDP on TNBC and explore the potential mechanisms.

**Methods:**

The efficacy and mechanisms of single or combined treatment were evaluated using Cell Counting Kit-8 (CCK8) assay, Annexin V-fluorescein isothiocyanate (FITC)/propidium iodide (PI) staining, wound healing assay, trans-well invasion/migration assay, hematoxylin-eosin (HE) staining, immunohistochemical (IHC) staining, Western Blot (WB) analysis, and fluorescence-activated cell sorting (FACS). An orthotopic model of TNBC was used to assess the *in vivo* treatment efficacy of single or combination treatment.

**Results:**

APS significantly enhanced the anti-proliferative, anti-migratory, and anti-invasive effects of DDP on TNBC cells. The combination of APS and DDP downregulated anti-apoptotic genes (Bcl2 and Bcl-xL) while upregulating pro-apoptotic genes (Puma, Cle-Caspase3, Cle-PARP), leading to enhanced apoptosis. This combination treatment increased E-cadherin levels, decreased Vimentin, Snail, Slug, and Twist levels, and effectively suppressed epithelial-mesenchymal transition (EMT)-associated cell invasion. In the orthotopic model of TNBC, a synergistic reduction in tumor growth was observed in mice treated with APS and DDP. Additionally, the combination of APS and DDP induced the infiltration of CD8^+^ T lymphocytes into the tumor immune microenvironment.

**Conclusion:**

The combination of APS and DDP exhibits more potent tumor inhibition and anti-tumor immunity than either agent alone, representing a novel approach to enhance therapeutic efficacy without increasing the side effects of DDP.

## Introduction

Breast cancer (BC) represents a major cause of cancer-related mortality in females, with an incidence of 2.26 million in 2020 [1]. BCs are categorized into hormone receptor-positive (HR^+^), human epidermal growth factor receptor 2 (HER2)-enriched, and TNBC [2], each exhibiting distinct differences in prognosis and treatment response. Among these subtypes, TNBC progresses the most rapidly and stands as the most heterogeneous molecular variant among BCs [3,4]. Despite significant advancements in treating advanced TNBC over the past decades, the prognosis remains unsatisfactory, with chemotherapy as the primary therapeutic approach against TNBC [5].

Cisplatin (DDP)-based chemotherapy, a widely utilized treatment for solid tumors, is recommended for TNBC treatment according to National Comprehensive Cancer Network (NCCN) guidelines [6]. DDP exerts its anti-tumor effects by inducing DNA double-strand breaks and facilitating apoptosis [7]. While low-dose DDP demonstrates minimal toxicity and side effects, its efficacy is compromised, and it is prone to drug resistance in TNBC treatment [8]. On the other hand, high doses of DDP lead to severe toxic side effects that patients find intolerable. Hence, additional research is needed to investigate synergistic interactions with DDP that could potentially overcome drug resistance and augment efficacy, while preserving the potential to enhance therapeutic outcomes in TNBC.

Non-toxic herbal medicines offer a promising alternative. APS is one of the important active components of *Astragalus membranaceus*, which has shown a variety of biological functions, including antioxidant, anti-inflammatory, anti-tumor, and immune regulation [9]. APS alone or in combination with cisplatin can enhance the chemotherapeutic and radiotherapeutic effects on various malignant tumors, such as non-small cell lung cancer [10], gastric cancer [11], and nasopharyngeal carcinoma [12]. Furthermore, APS has the great potential to reduce toxic side effects of chemo-drugs, such as reducing cisplatin nephrotoxicity [13], and epirubicin cardiotoxicity [14]. APS plays a crucial role in reshaping the immune microenvironment and increasing the sensitivity of anti-tumor treatment [15,16]. Recently, it has been reported that APS was potent in inhibiting breast cancer development by facilitating macrophage polarization towards the M1 type [17,18]. This study assessed the impact of combining APS and DDP treatments on the proliferation, apoptosis, and invasion ability of TNBC. Additionally, we examined the infiltration of CD8^+^ T lymphocytes in the tumor immune microenvironment to shed light on potential immunomodulatory outcomes.

## Materials and Methods

### Chemicals and reagents

APS (Cat No. A7970, purity ≥90%) was purchased from Solarbio (Beijing, China), and DDP (Cat No. HY-17394, purity 99.84%) was obtained from MedChemExpress (Monmouth Junction NJ, USA). Cell proliferation assay (CCK8) kit (Cat No. KGA9305-1000) and Annexin V-fluorescein isothiocyanate (FITC)/propidium iodide (PI) Apoptosis Detection Kit (Cat No. KGA1102-100) were sourced from KeyGen Biotech. (Nanjing, China). A Sodium dodecyl sulfate-polyacrylamide gel electrophoresis (SDS-PAGE) kit (Cat No. G4842) was acquired from Solarbio. Polyvinylidene fluoride membranes (PVDF, Cat No. 03010040001) were obtained from Millipore Corp (Bedford, MA, USA). Primary antibodies against CD8 (Cat No. K002374P), Bcl-xL (Cat No. K001595P), and Caspase3 (Cat No. K009430M) were acquired from Solarbio. Primary antibodies against PARP (Cat No. SAB4500487), Bcl2 (Cat No. SAB4500003), E-cadherin (Cat No. SAB5700789), p21 (Cat No. SAB5700189), Vimentin (Cat No. SAB4503083), Puma (Cat No. SAB5701486), Snail (Cat No. SAB5700796), Slug (Cat No. SAB5700672), Twist (Cat No. SAB5701071) and β-Actin (Cat No. SAB5600204) were purchased Sigma-Aldrich (St. Louis, MO, USA), Horseradish peroxidase (HRP)-conjugated goat anti-mouse (Cat No. ab205719) and goat anti-rabbit (Cat No. ab205718) IgG secondary antibodies were acquired from Abcam (Cambridge, UK), The rabbit specific HRP/DAB Detection immunohistochemical (IHC) Kit (Cat No. ab64259) was purchased from Abcam, FITC-CD3 (Cat No. ab239226), and APC-CD8 (Cat No. ab288234) were obtained from Abcam. NP40 lysis buffer (Cat No. P0013F) and RIPA lysis buffer (Cat No. P0013E) were purchased from Beyotime (Shanghai, China). NP-40 lysis buffer contains 50 mM Tris-HCl (pH 7.4), 150 mM NaCl, 1% NP-40, and 5 mM EDTA. RIPA lysis buffer contains 25 mM Tris-HCl (pH 7.6), 150 mM NaCl, 1% NP-40, 1% sodium deoxycholate, and 0.1% SDS.

### Cell line and cell culture

The murine TNBC 4T1 cell line (obtained from the Cell Bank/Stem Cell Bank, Chinese Academy of Sciences, Shanghai, China) was cultured in Dulbecco’s modified Eagle’s medium (DMEM, KeyGen Biotech) supplemented with 10% fetal bovine serum (FBS, Roche, Basel, Switzerland) and 1% penicillin/streptomycin (KeyGen Biotech.) at 37°C in a humidified incubator supplied with 5% CO_2_.

### Cell proliferation assay

The 4T1 cells were cultured in 96-well plates at a density of 5 × 10^3^ cells per well and incubated for 24 h to reach the logarithmic growth phase. Subsequently, drug treatments were administered at predetermined intervals. Concentrations of APS (0, 1.25, 2.50, 5, 10, and 20 mM) [15,19] and DDP (0, 2.50, 5, 10, 20, 40, 80 μM) [20] were chosen based on previous studies. Cell proliferation was examined using the CCK8 cell proliferation assay kit according to the manufacturer’s protocol. Optical density (OD) values were measured at 450 nm using an absorbance microplate reader (BioTek, Hercules, CA, USA).

### Detection of apoptosis via annexin V-FITC/PI staining

The 4T1 cells (1 × 10^5^ cells/well) were seeded in six-well plates and cultured for 24 h. Subsequently, cells were treated with APS (2.50 mM), DDP (5 μM), or a combination of APS and DDP (2.50 + 5 μM) for an additional 24 h. After treatment, cells were harvested, washed twice with phosphate-buffered saline (PBS), and stained with the Annexin V-FITC/PI staining kit in binding buffer for 30 min at room temperature in the dark. The labeled cells were sorted and counted using a FACScan Cytometer (BD Biosciences, San Jose, CA, USA), and the percentage of apoptotic cell death was analyzed using FlowJo software.

### Wound healing assay

The 4T1 cells (3 × 10^5^ cells/well) were seeded in 12-well plates until reaching 100% confluence. A single scratch was made in the center of each well using a 10 μL sterile pipette tip. Cells were then rinsed with PBS to remove floating cells and treated with APS (2.50 mM) and/or DDP (5 μM). Wound closure was observed at 0, 12, and 24 h using an optical inverted microscope (Zeiss, Germany, Magnification: ×100), and the width was measured with Image J software. The relative area compared with the untreated control group was quantified.

### Transwell migration and invasion assay

For the transwell migration assay, 4T1 cells (2 × 10^5^ cells/mL) were treated with APS (2.50 mM), DDP (5 μM), or a combination of APS (2.50 mM) and DDP (5 μM) for 6 h. Subsequently, the cells were seeded into the upper chambers of the transwell plates (24-well inserts; diameter, 6 mm; pore size, 8 µm; Corning Life Sciences, Shanghai, China), while the lower chambers contained a complete DMEM medium with 20% FBS. After 24 h, the migrated cells were stained with crystal violet (Cat No.: C0775, Sigma Aldrich) and counted using an inverted light microscope (Carl Zeiss, Oberkochen, Germany, magnification: ×200).

For the transwell invasion assay, the transwell plate membranes were precoated with matrigel (Cat No.: 356234, Solarbio) for 4 h at 37°C. The subsequent steps were identical to the Transwell migration assay.

### Animal studies

Six-week-old female B alb/c mice (~16–18 g) were purchased from the Animal Center of Xuzhou Medical University. All animals were housed in the Animal Center under a specific pathogen-free (SPF) environment with *ad libitum* access to food and water, maintaining a 12:12 light-dark (LD) cycle at temperatures between 22°C–25°C and humidity between 30%–50%. Ethical approval for all animal experiments was obtained from the Ethics Committee of Xuzhou Medical University Ethical Code: 202203A092.

The mouse model of TNBC was established by orthotopic injection of 4T1 cells (1 × 10^4^ cells/μL) into the mammary fad pat at the base of the 2nd thoracic nipple. Eight days after cell inoculation, tumor-bearing mice were randomly assigned to receive treatment with vehicle solution (PBS), APS (100 mg/kg), DDP (3 mg/kg), or the combination of APS and DDP (n = 8 mice/group) via intraperitoneal (i.p.) injection. APS was administered every 2 days and DDP was administered every 4 days. Tumor growth was measured externally every 2 days using vernier calipers, and the tumor volume was calculated using the formula: length × width^2^ × 0.52. The body weight of the mice was also recorded every 2 days. At the study endpoint, the mice were euthanized, and tumors along with major organs were excised for histological analyses.

### CD8^+^ T cell infiltration in tumor issues

Fresh tumor samples (30 mm^3^) from B alb/c mice were finely minced in 2 mL tissue storage solution (Cat No.: 130-100-008, Miltenyi Biotech. Bergisch-Gladbach, Germany), and cells were dissociated through blunt dissection. A 150 μL cell suspension was then transferred to EP tubes and centrifuged at 2000 rpm for 5 min. The resulting pellet was stained with a fluorescence-coupled antibody mixture (FITC-CD3 at 1:300, APC-CD8 at 1:300) at 50 μL per tube. Staining was performed at 4°C, and after 30 min, 1 mL of PBS was added to stop staining. Following centrifugation (2000× *g*) for 3 min, the supernatant was discarded, and the sample was washed with 1 mL of PBS. The samples were then resuspended in 300 μL of PBS, thoroughly mixed, and subjected to automated testing. Data analysis was performed using FlowJo software.

### Hematoxylin and eosin (HE) and IHC analysis

Tumor tissues, lung, liver, and kidney samples were extracted from B alb/c mice and fixed in 4% paraformaldehyde, followed by dehydration and embedding in paraffin. Paraffin-embedded samples were sectioned and mounted on microscope slides (4 μM thick). HE staining was performed as previously described [21].

For IHC analysis, sections were deparaffinized, rehydrated, and subjected to antigen retrieval using citric acid tissue antigen retrieval solution at high temperatures. Endogenous peroxidase reactivity was blocked by applying 3% H_2_O_2_. Sections were then incubated overnight at 4°C with respective primary antibodies (Ki67 at 1:4000, P21 at 1:200, Puma at 1:400, CD8 at 1:500, and Bcl2 at 1:300, diluted in 10% goat serum). After washing with PBS 3 times, sections were incubated with secondary antibodies at 37°C for 20 min. Following another PBS wash, a DAB staining solution was added, and staining was stopped with ddH_2_O upon observation of a brown precipitate under an optical microscope. Finally, sections were counterstained using hematoxylin, dehydrated, and fixed. Images at a magnification of ×200 were captured using an inverted light microscope (Olympus, Japan). Two pathologists independently assessed the HE and IHC staining results to examine alterations in both cellular morphology and the surrounding tissues. Quantitative analysis of Ki67, Bcl2, P21, Puma, and CD8 was performed as described previously [22–26].

### Western blot analysis

For cell samples, treated 4T1 cells were harvested and lysed using NP40 buffer. For tissue samples, tumors (30 mm^3^) obtained from mice were added to 200 μL RIPA lysate buffer and crushed using an ultrasonic crusher. Protein concentrations were determined using the Coomassie brilliant blue protein assay (Cat No. ST1119, Beyotime, China). Equal amounts of protein (20 µg) were separated by SDS-PAGE and transferred onto a PVDF membrane. After blocking with 5% nonfat dry milk in PBST buffer for 30 min, the membranes were incubated with primary antibodies overnight at 4°C, followed by probing with secondary antibodies. Subsequently, protein bands were detected using an ECL kit (Cat No. WBULP, Millipore, MA, USA) and ImageJ software was used for protein band quantification.

### Statistical analysis

All experiments were independently conducted thrice to ensure the reliability and reproducibility of the results. Statistical analyses were performed using GraphPad Prism 8.0 software. Data were presented as mean ± SD. Differences between treatment and control groups were analyzed using one-way analysis of variance (ANOVA). *Post hoc* tests for multiple comparison analyses were conducted using the Tukey method. Statistical significance was defined as *p < 0.05, indicating significance, while **p < 0.01 and ***p < 0.001 were considered highly significant. These statistical thresholds were applied to determine the significance level of the study.

## Results

### The combined treatment of APS and DDP effectively suppresses proliferation and induces apoptosis in TNBC (4T1) cells compared to individual treatments alone

**Figure 1:**
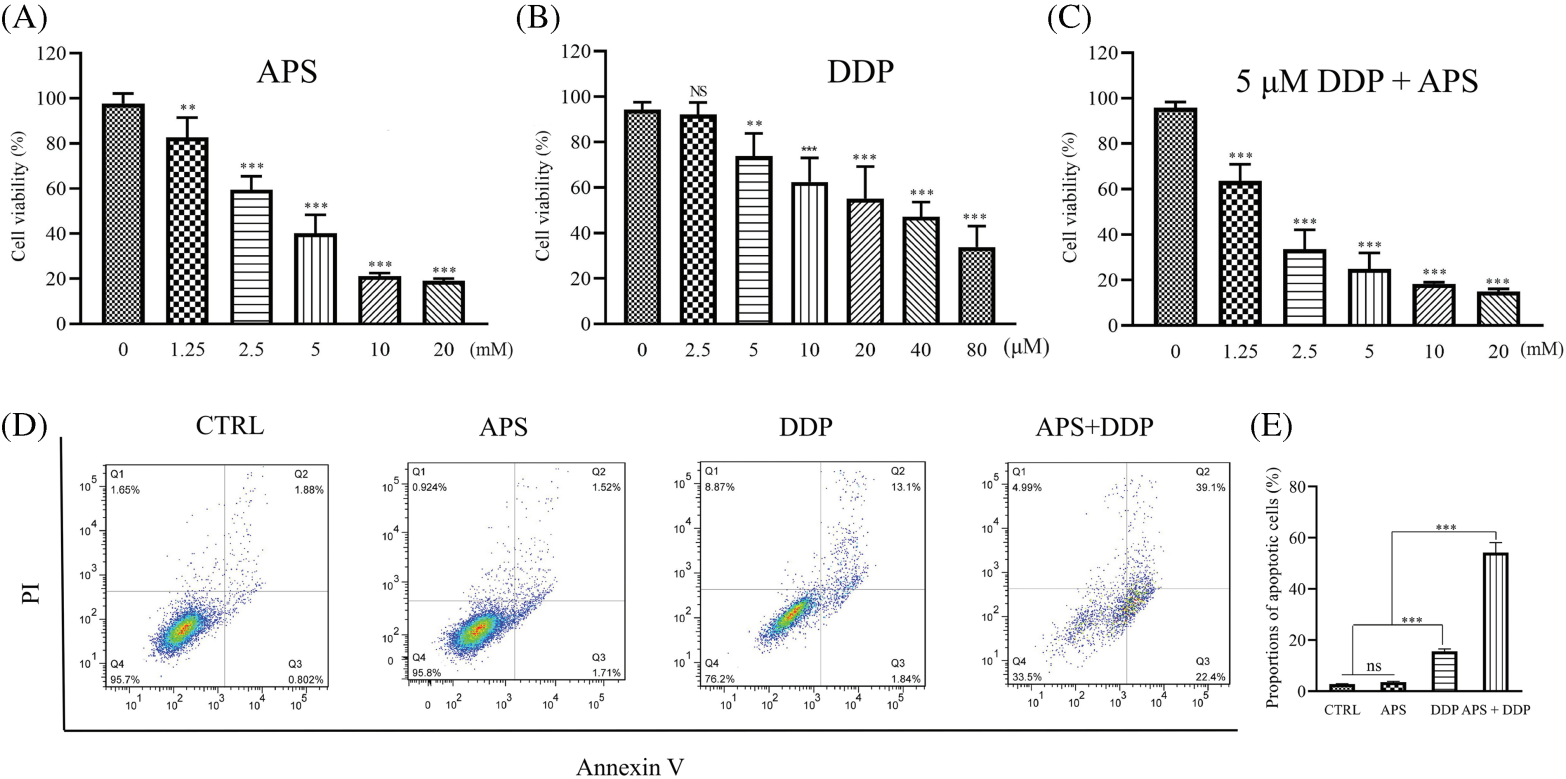
Effect of APS and/or DDP on 4T1 cell proliferation and apoptosis. (A–C) Proliferation of cells treated with different concentrations of APS (A), DDP (B), and APS combined with DDP (5 μM) (C) was determined using CCK8 assay. Quantitative analysis of cell proliferation expressed as a ratio of the control group. (D) Cell apoptosis treated with APS, DDP, and APS + DDP was determined using Annexin V-FITC/PI assay. (E) Quantitative analysis of cell apoptosis expressed as a percentage of the control group. ns: no significance, **p < 0.01, ***p < 0.001.

To evaluate the efficacy of APS and DDP, alone or in combination on the proliferation of 4T1 cells, we initially conducted a CCK8 assay *in vitro*. As depicted in Fig. 1A, treatment with different concentrations of APS in 4T1 cells resulted in varying degrees of inhibition. Notably, significant inhibition of 4T1 cell proliferation was observed at 1.25 mM APS, with a more pronounced effect observed with increasing doses. However, at concentrations of 10 and 20 mM, no significant difference in inhibitory effects was observed. The IC50 value of APS was 3.46 mM.

DDP was also demonstrated to effectively inhibit the proliferation of 4T1 cells, as illustrated in Fig. 1B. Notably, 4T1 cells were significantly inhibited by DDP at a concentration of 5 µM. The IC50 value of DDP was 7.99 µM. To highlight the synergistic effect of APS on DDP, we subsequently selected a concentration of 5 µM for combination studies. Combining different concentrations of APS with DDP (5 µM) resulted in significant proliferation inhibition, particularly notable at the combination of 2.50 mM APS and 5 µM DDP (Fig. 1C).

Next, we determined the effects of single or combination treatments on the apoptosis level of 4T1 cells using a flow cytometry assay. A low level of apoptosis was observed with a single treatment using either APS or DDP, as depicted in Figs. 1D and S1. However, when combined with APS (2.50 mM) and DDP (5 µM), the apoptosis level was more pronounced compared to the control, APS-only, or DDP-only groups (Fig. 1E). Therefore, we selected a specific combination (2.50 mM APS + 5 µM DDP) for subsequent *in vitro* experiments.

### The combined treatment of APS and DDP effectively suppresses cell migration and invasion in TNBC (4T1) cells compared to individual treatments alone

**Figure 2:**
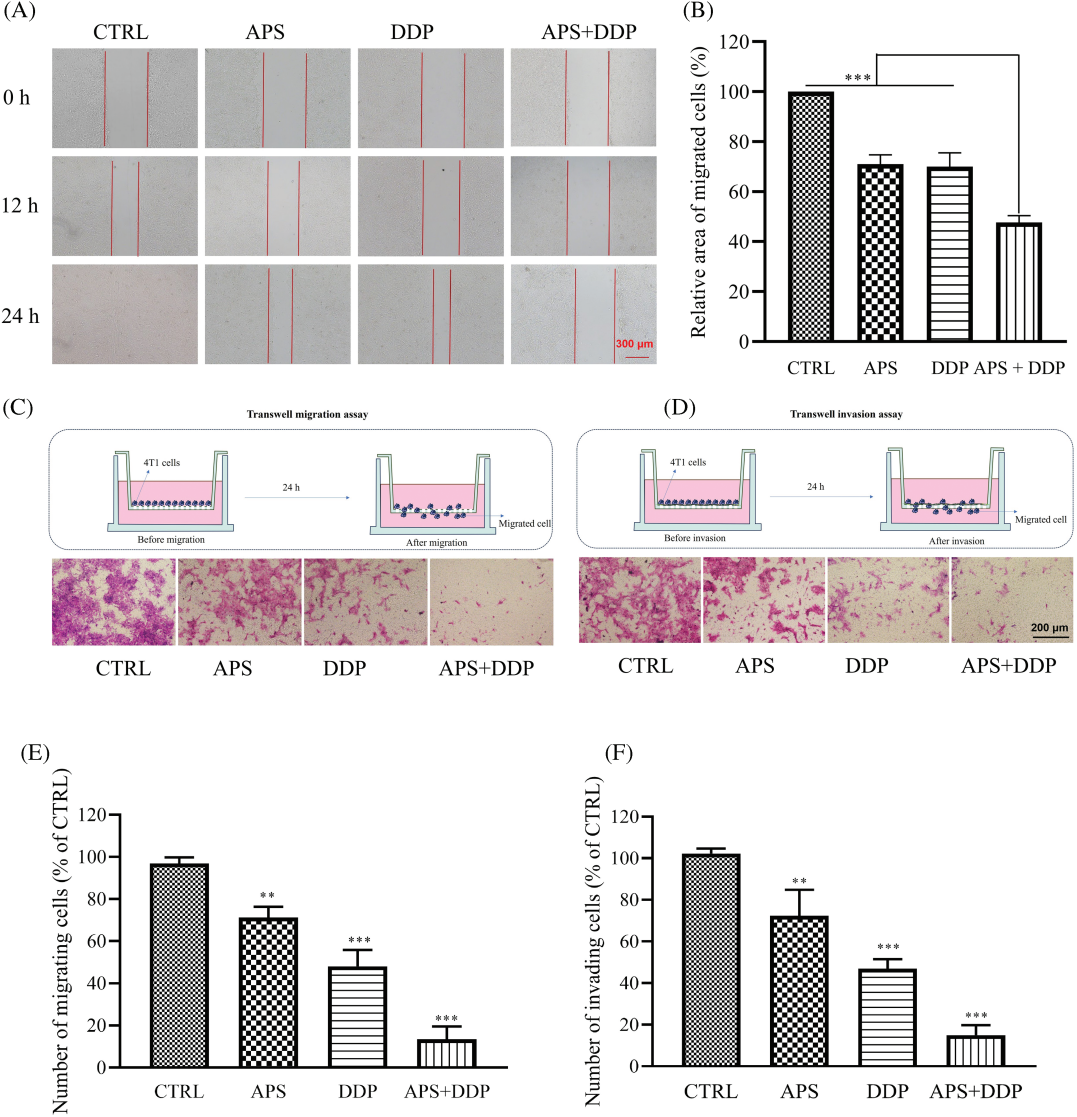
Effect of APS and/or DDP on 4T1 cell migration and invasion. (A) Cells were scratched and subsequently treated with different drugs (APS and/or DDP). Representative images were acquired at 0, 12, and 24 h. Magnification, ×100. (B) Quantitative analysis of cell migration expressed as a percentage of the control group (n = 3). (C) Cells were treated for 24 h. Migration ability was determined using a Transwell migration assay. Magnification, ×200. (D) Cells were treated for 24 h. Invasive ability was determined using a Transwell matrigel assay. In (E and F), quantitative results from migration and invasion assays are shown in the low panels (n = 3). **p < 0.01, ***p < 0.001.

Cell migration is closely associated with epithelial-mesenchymal transition (EMT). We conducted wound-healing experiments to investigate the potential differential effects of APS and DDP, alone or combined together on 4T1 cell migration. Remarkably, the combined drug treatment demonstrated significant inhibition of 4T1 cell migration. Following treatment with APS, DDP, or the combination of APS and DDP for 12 h, differences in their migration began to manifest. The most notable divergence was observed at 24 h, where scratches in the APS or DDP group exhibited a relatively apparent healing state. However, in the combined APS and DDP group, the scratches’ healing ability was significantly inhibited (Fig. 2A,B). Further examination of 4T1 cell migration through transwell migration assays revealed a significant reduction in the number of cells passing through the transwell pores in the combination group compared to monotherapy, indicating that the combined use of drugs can substantially inhibit the migration ability of TNBC (Fig. 2C,E).

Invasion, a crucial aspect of EMT, was further examined through transwell invasion experiments. While both APS and DDP individually exhibited varying degrees of 4T1 cell invasion inhibition compared to the CTRL group, the combined APS + DDP group demonstrated more pronounced inhibition of invasion (Fig. 2D,F).

### The combined treatment of APS and DDP altered the protein profile associated with apoptosis and EMT in TNBC cells

**Figure 3:**
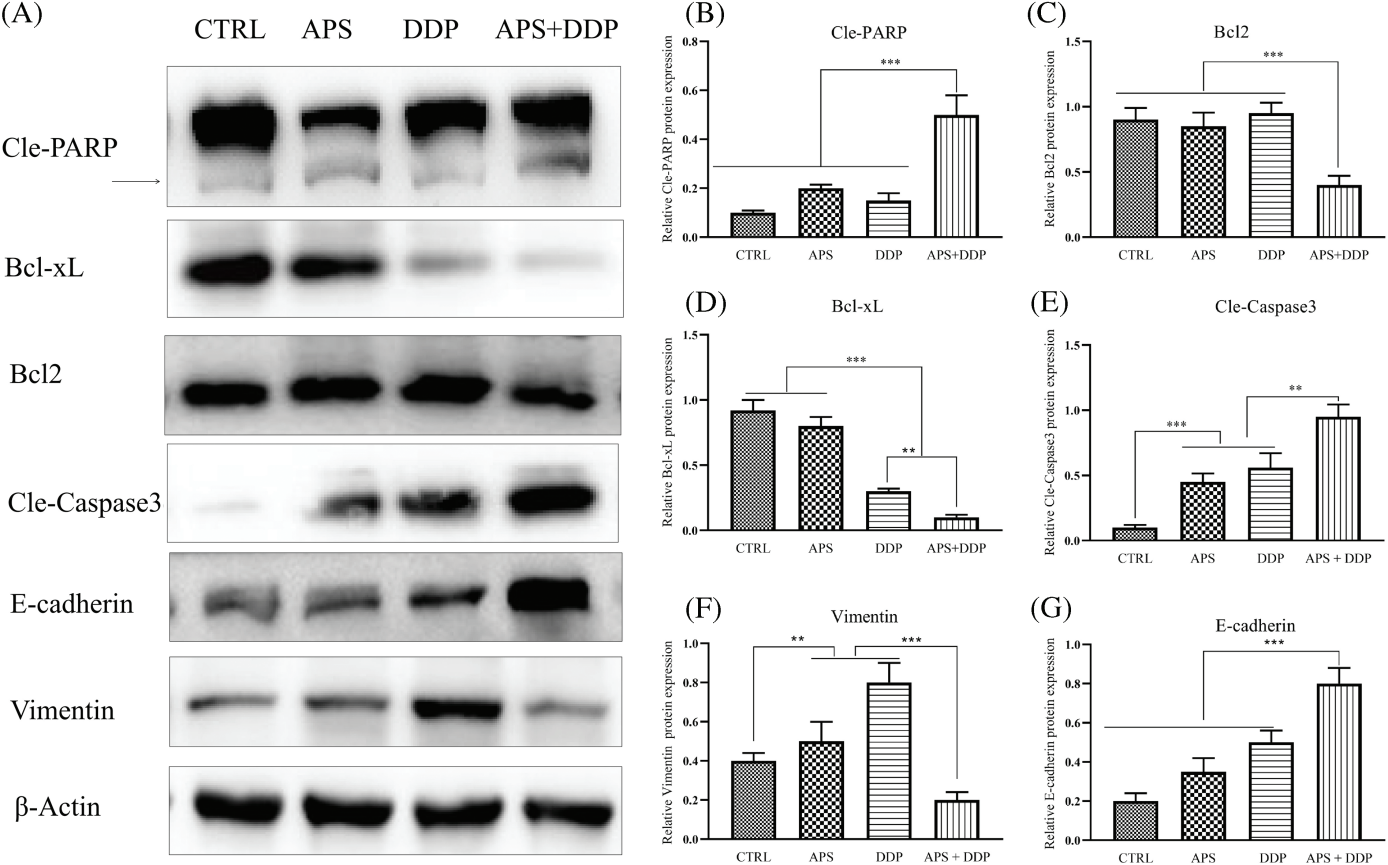
Effect of APS and/or DDP on 4T1 cell apoptosis and EMT in TNBC (4T1) cells. (A) Representative protein levels of Cle-PARP, Bcl-xL, Bcl2, Cle-Caspase3, E-cadherin, and Vimentin were assessed using Western blot. (B–G) Quantitative results of protein levels of Cle-PARP, Bcl2, Bcl-xL, Cle-Caspase3, E-cadherin, and Vimentin (n = 3). **p < 0.01, ***p < 0.001.

To elucidate the mechanisms underlying the suppression of cell proliferation and induction of apoptosis in breast cancer cells by APS, DDP, and the combination of APS and DDP, we analyzed apoptosis-related proteins (Cle-PARP, Cle-Caspase3, Bcl2, and Bcl-xL). As depicted in Fig. 3A–E, the expression levels of Bcl-xL and Bcl2 were lower in cells treated with the combination of APS and DDP compared to those treated with either APS or DDP alone (Fig. 3C,D). In contrast, Cle-PARP and Cle-Caspase3 were significantly upregulated in the combined treatment group (Fig. 3B,E). We further investigated the impact on the levels of EMT-associated molecules (E-cadherin, Vimentin, Snail, Slug, and Twist). The combination of APS and DDP upregulated E-cadherin expression and downregulated the levels of Vimentin, Snail, Slug, and Twist (Figs. 3F,G and S2). Collectively, these results indicate that the combination of APS and DDP has the potential to promote apoptosis and inhibit cell invasion of 4T1 cells more effectively than treatment alone.

### The combination of APS and DDP produces a strong synergistic inhibitory effect on TNBC in mice

**Figure 4:**
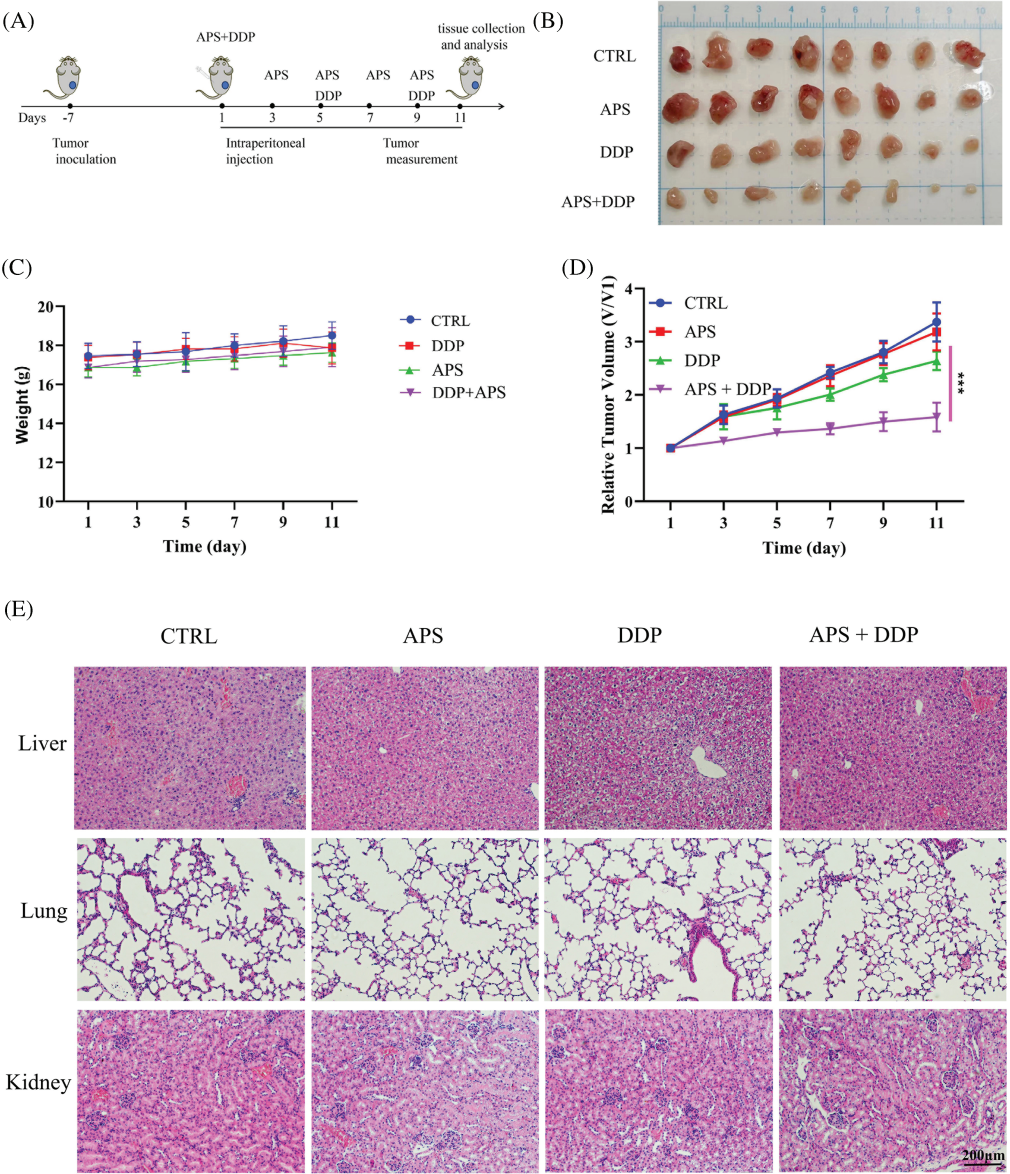
APS enhances the antitumor activity of DDP in 4T1 tumors. (A) Schedule of tumor implantation, APS, DDP, and APS + DDP injection, tumor measurement, tissue collection, and analysis. (B) Photographs of tumors on day 18. (C) Body weight loss of tumor-bearing mice after different treatments. (D) Growth curves of tumors in 4T1 tumor-bearing mice after intraperitoneal (i.p.) injections of PBS, APS (100 mg/kg), DDP (3 mg/kg), APS + DDP (100 + 3 mg/kg) (n = 8 mice/group). (E) Representative HE staining images of CTRL, APS, DDP, and APS + DDP group treated tumor-bearing B alb/c mouse sacrificed on day 18. ***p < 0.001.

During *in vivo* experiments, a TNBC mouse model with subcutaneous tumors in the third mammary gland was established using 4T1 cells, and the mice were treated with APS, DDP, and the combination of APS and DDP according to Fig. 4A. After eighteen days, the mice were euthanized to evaluate the anti-cancer effects of single and combination treatments. As shown in Fig. 4B, APS + DDP effectively inhibited tumor growth. Notably, subcutaneous tumors in mice exhibited resistance to APS alone, and while the DDP group showed a mild inhibitory effect on tumor tissue growth, it did not reach statistical significance (*p* > 0.05 *vs*. control group). However, APS + DDP demonstrated significantly more effective tumor inhibition (*p* < 0.001). The tumor volume in the APS + DDP group was the lowest, indicating a potent anti-tumor effect (Fig. 4D). To assess the safety of the treatment strategies, we measured the body weight of mice and examined major organs (lung, liver, and kidney) using HE staining. The body weight remained comparable (Fig. 4C), and mice exhibited good tolerance to the drugs (Fig. 4E). No significant differences in organ changes were observed among all groups, suggesting a favorable safety profile for the treatment.

**Figure 5:**
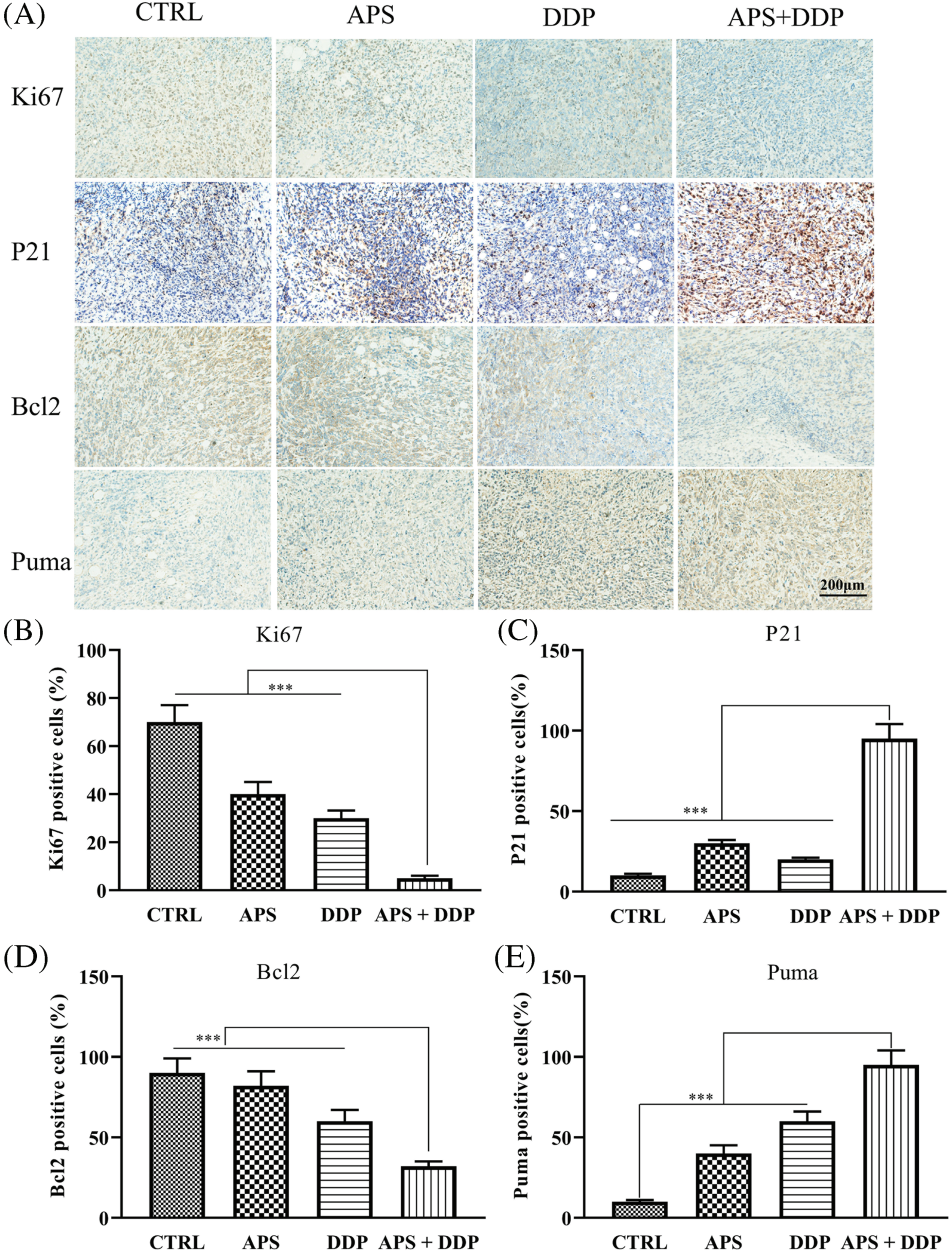
*In vivo* evaluation of the effects of APS and DDP, alone or in combination, on proliferation and apoptosis. (A) The protein expression levels of Ki67, Bcl2, p21, and Puma were assessed using IHC. (B–E) The protein expression levels of Ki67, Bcl2, p21, and Puma were quantified. ***p < 0.001.

Next, we determined whether APS, DDP and their combination decreased Ki67 levels *in vivo*. The levels of Ki67 (Fig. 5A) in tumor tissues from different groups of mice were examined using IHC. Our findings revealed that, unlike the control, APS, and DDP groups, the mice treated with both APS and DDP demonstrated downregulation of Ki67 *in vivo* (Fig. 5B). These data suggest APS may improve the chemo-sensitivity of tumor tissues by inhibiting proliferation. Additionally, we explored the expression of p21 in different groups using IHC (Fig. 5A), and the results indicated an increased p21 in the combined group (Fig. 5C).

Furthermore, we investigated whether APS and DDP, alone or in combination altered the expression of apoptosis-associated proteins. The expression levels of Bcl2 and Puma (Fig. 5A,D,E) in tumor tissues from different groups of mice were assessed using IHC. Our findings demonstrated that, unlike the control group, the combination of APS and DDP downregulated Bcl2 and upregulated Puma levels *in vivo*. This comprehensive modulation contributed to induced apoptosis and increased chemo-sensitivity of TNBC.

### The combination of APS and DDP increases the CD8^+^T lymphocyte infiltration in TNBC

**Figure 6:**
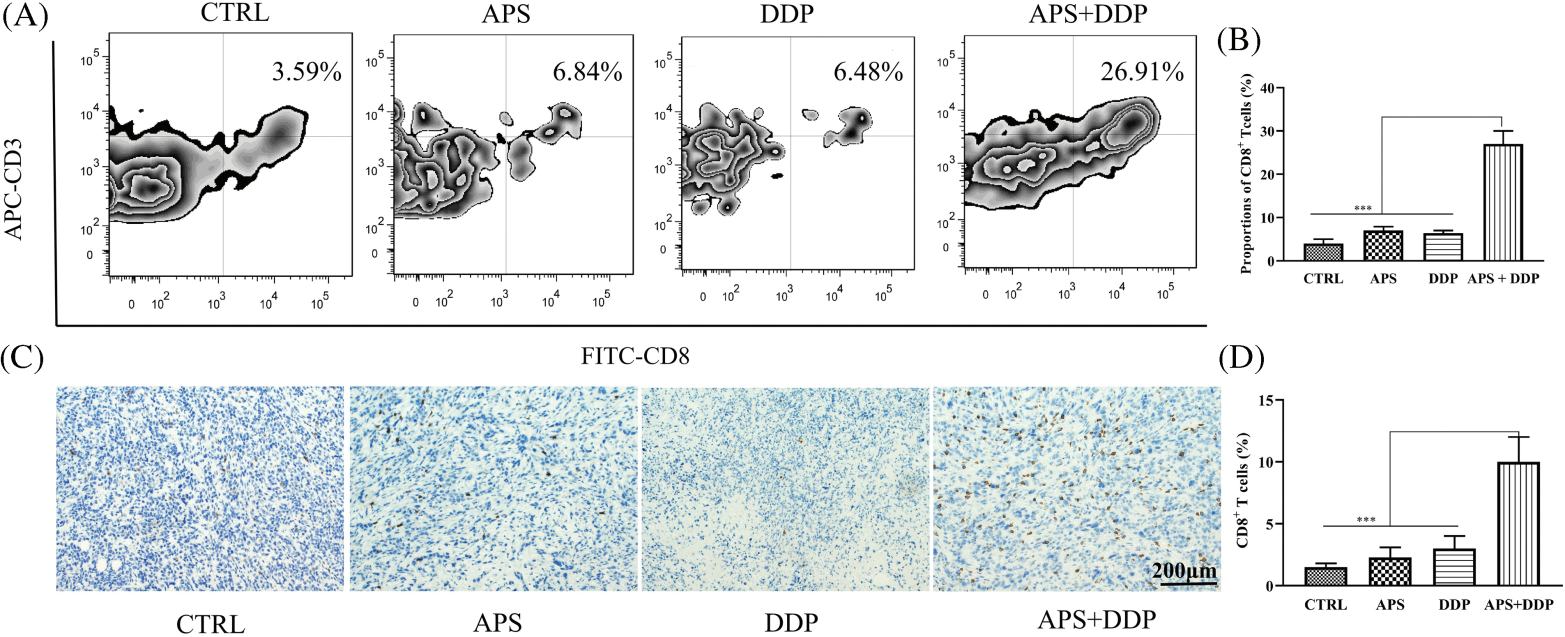
Evaluation of CD8^+^ T infiltration in tumor tissues from mice treated with APS, DDP, alone or in combination. (A) The CD8^+^ T expression levels of different groups were assessed using FACS. (B) The CD8^+^ T infiltration levels were quantified. (C) The protein expression levels of CD8 were assessed using IHC. (D) The CD8^+^ T cells (%) were quantified. ***p < 0.001.

APS is known to play a role in regulating immunity, but the mechanisms by which APS modulates human immunity require further exploration. In this study, fluorescence-activated cell sorting (FACS) revealed a significant increase in the proportion of CD8^+^ T cells in the combined group compared to the APS and DDP groups (Fig. 6A,B, *p* < 0.001). Subsequently, we conducted IHC to assess the protein expression of CD8 in different groups. Our findings uncovered upregulation of CD8 protein expression in the combined group, which demonstrated statistical significance compared to the control group and APS and DDP groups (Fig. 6C,D).

## Discussion

With a deeper understanding of the molecular classification of TNBC, there are more therapeutic options for TNBC available, such as chemotherapy, immunotherapy, and targeted therapy. However, the efficacy is still very unsatisfactory [27]. Studies have confirmed that APS can improve the efficacy of anti-tumor therapy, reduce the side reactions of anti-tumor therapy, reverse drug resistance, and regulate humoral and cellular immunity [9]. In this study, we evaluated the effectiveness of a combination of APS and DDP on TNBC and explored the potential mechanisms, including apoptosis induction, proliferation inhibition, invasion and metastasis inhibition, and increased infiltration of CD8^+^ T cells in TNBC.

Proliferation is a crucial factor in predicting breast cancer prognosis and treatment response [28]. Ki67 is a non-histone nuclear protein associated with cellular proliferation, and its proliferation index has been correlated with poor clinical prognosis. A higher Ki67 index is indicative of a worse prognosis [29]. In our *in vitro* experiments, the CCK8 assay was employed for 4T1 cell growth assessment in different groups, revealing a stronger inhibition of cell proliferation in the combined group. In the *in vivo* experiments, IHC was utilized to detect the expression level of Ki67, showing a decrease in proliferation in the combined group. Further assessment of p21 protein expression by IHC demonstrated a significant increase in the combined group. P21, known for binding to various cell cycle cyclins, induces cell cycle arrest and consequently inhibits cell proliferation [30]. The observed correlation between increased p21 expression and downregulated Ki67 levels substantiated cell growth inhibition following the combination treatment of APS and DDP treatment.

Apoptosis, a programmed mode of cell death, plays a crucial biological role, and dysregulation in apoptotic cell death machinery is a hallmark of cancer [31]. In our present *in vitro* studies, Annexin V-FITC/PI double staining indicated a significantly higher apoptosis ratio in the combined group than in the mono-drug and control groups. At the cellular level, Western blot results showed downregulation of Bcl2 and Bcl-xL expression and upregulation of Cle-Caspase3 and Cle-PARP expression in the combined group. *In vivo* experiments using IHC revealed decreased Bcl2 expression and upregulated Puma expression in the combination group. These proteins play distinct roles in different stages of the mitochondrial apoptosis pathway. The Bcl2 family, with members such as Bcl2 and Bcl-xL, regulates mitochondrial apoptosis by influencing the location of various molecules on the mitochondrial membrane [32]. Caspase3, a member of the cysteine aspartate protease family, is essential in apoptosis by mediating the cleavage of specific target proteins [33]. It is considered a key enzyme in the apoptotic pathway. During the early stages of apoptosis, activated caspase3 undergoes cleavage into two small subunits, ultimately leading to the initiation of apoptosis. Consequently, caspase3 is commonly utilized as an early detection indicator of cell apoptosis [34]. PARP (poly(ADP-ribose) polymerase) is a Zn^2+^-dependent eukaryotic DNA binding protein with the ability to specifically recognize DNA break ends, making it an essential DNA repair enzyme [35]. In early apoptosis, activated caspase3 acts on PARP, resulting in its cleavage into 89 kD and 24 kD fragments. The loss of PARP’s repair function occurs as the 89 kD fragment migrates out of the nucleus. Therefore, the 89 kD fragment serves as a valuable indicator for detecting apoptosis. The significant cleavage expression of PARP observed in our present study further supports the occurrence of apoptosis in response to the treatment with APS and DDP combination. Our study has demonstrated the effect of APS and DDP treatment in down-regulating Bcl2. Accumulating evidence has shown that Bcl2 can precisely regulate the calcium level in the mitochondria by directly interacting with IP3R and indirectly modulating IP3R [36], thereby controlling apoptosis, which makes targeting Bcl2 an attractive anticancer strategy (REF). However, the role of APS on calcium homeostasis and ER stress of tumor cells warrants further investigation.

TNBC is highly prone to metastasis, a critical factor contributing to uncontrollable tumor growth [37]. Therefore, it is crucial to select clinical drugs that can inhibit the metastasis and invasion ability of tumors. The combination of APS and DDP is expected to enhance the inhibition of invasion and metastasis. In our study, we explored the effects of APS plus DDP on the invasion and metastasis of 4T1 cells, which are epithelial-derived TNBC cells. EMT initiation represents a crucial step in the invasion and metastasis of these cells [38].

During the process of EMT, alterations occur in various signaling pathways and transcription factors. The decrease in E-cadherin expression in epithelial cells contributes to invasion and metastasis, while the increased expression of Vimentin further induces an epithelial cell phenotype, facilitating cell invasion and metastasis [39]. Snail, Slug, and Twist are well-defined EMT markers. These proteins act as transcription factors to bind to the E-box and downregulate the expression of E-cadherin, thereby promoting tumor migration and invasion [40]. Reversing this EMT state can lead to a decline in the invasion and metastasis ability of tumors, thereby improving treatment outcomes. Herein we explored the invasion and metastasis effects of APS plus DDP on 4T1 cells. The wound healing experiment and transwell migration assay in the combined group revealed slowed or incomplete scratch healing, indicating weakened migration of TNBC cells. In the subsequent transwell invasion assay, the combined group exhibited a reduction in tumor cells penetrating the matrigel, suggesting a stronger inhibitory effect on invasion. Mechanistic insights were further explored through Western blot experiments, revealing a significant increase in E-cadherin expression and a significant downregulation of Vimentin, Snail, Slug, and Twist in the combined drug group. These results indicate that the combined treatment weakened the invasion and metastasis ability of 4T1 cells by influencing the expression of different invasive proteins. The findings emphasize the potential of the APS and DDP combination in mitigating the aggressive behavior of TNBC through the regulation of EMT-associated proteins.

Immunotherapy, particularly with immune checkpoint inhibitors (ICIs), has gained significant momentum in cancer treatment [41]. Nonetheless, poor responsiveness to ICIs is observed in some cases, categorically known as “cold” tumors [42]. One strategy to convert cold tumors into hot tumors is to enhance CD8^+^ T lymphocyte infiltration, thereby improving the efficacy against TNBC. The 4T1 cell line used in our study represents a poorly immunogenic breast cancer cell line [43]. Our FACS study and IHC examination revealed increased expression of CD8^+^ T lymphocytes in the drug combination group, suggesting that the integration of Chinese medicine and chemotherapy could be an effective approach to transform TNBC from a cold tumor to a hot tumor. This bold assumption prompts further exploration, suggesting that the addition of ICIs to Chinese medicine and chemotherapy may further enhance the treatment efficiency of TNBC. Nevertheless, this proposition requires validation through well-designed clinical tests.

Despite our notable findings, there are some limitations in our study. In the animal experiments, although more CD8^+^ T lymphocytes were observed to infiltrate the tumor tissue in the combined group, the specific mechanism leading to this increased infiltration was not thoroughly explored. Further investigations are warranted to elucidate the mechanism behind CD8^+^ T lymphocyte infiltration. In summary, our animal experiments suggest that the combination treatment has the potential to promote the transformation from “cold” to “hot” tumors. However, further verification is warranted to determine whether this change in the immune microenvironment can indeed translate into tangible benefits for immunotherapy.

## Conclusions

In summary, the combination of APS and DDP demonstrates more potent inhibitory effects on the proliferation and invasion capabilities of TNBC cells, while concurrently promoting apoptosis, compared with single treatment alone. Notably, this combination also enhances the infiltration of CD8^+^ T cells in TNBC, suggesting its potential to potentiate antitumor immunity and transform an immunologically “cold” tumor into a “hot” tumor.

## Supplementary Materials

Figure S1 Effect of APS and/or DDP on 4T1 cell apoptosis. Apoptosis of cells treated with APS, DDP, and APS + DDP was detected using Annexin V-FITC/PI assay.

Figure S2 Effect of APS and/or DDP on 4T1 cell EMT in TNBC (4T1) cells. Representative protein expression of Snail, Slug, and Twist were assessed using Western blot.

## Data Availability

The data are available from the corresponding author upon request.

## Supplementary Materials

The supplementary material is available online at https://doi.org/10.32604/or.2024.050057.
