# Supplementary figures and images for: Astragalus polysaccharide enhances the therapeutic efficacy of cisplatin in triple-negative breast cancer through multiple mechanisms
Source: Oncol Res. 2025 Feb 28;33(3):641–51. doi: 10.32604/or.2024.050057 (PMC11915043; doi:10.32604/or.2024.050057)

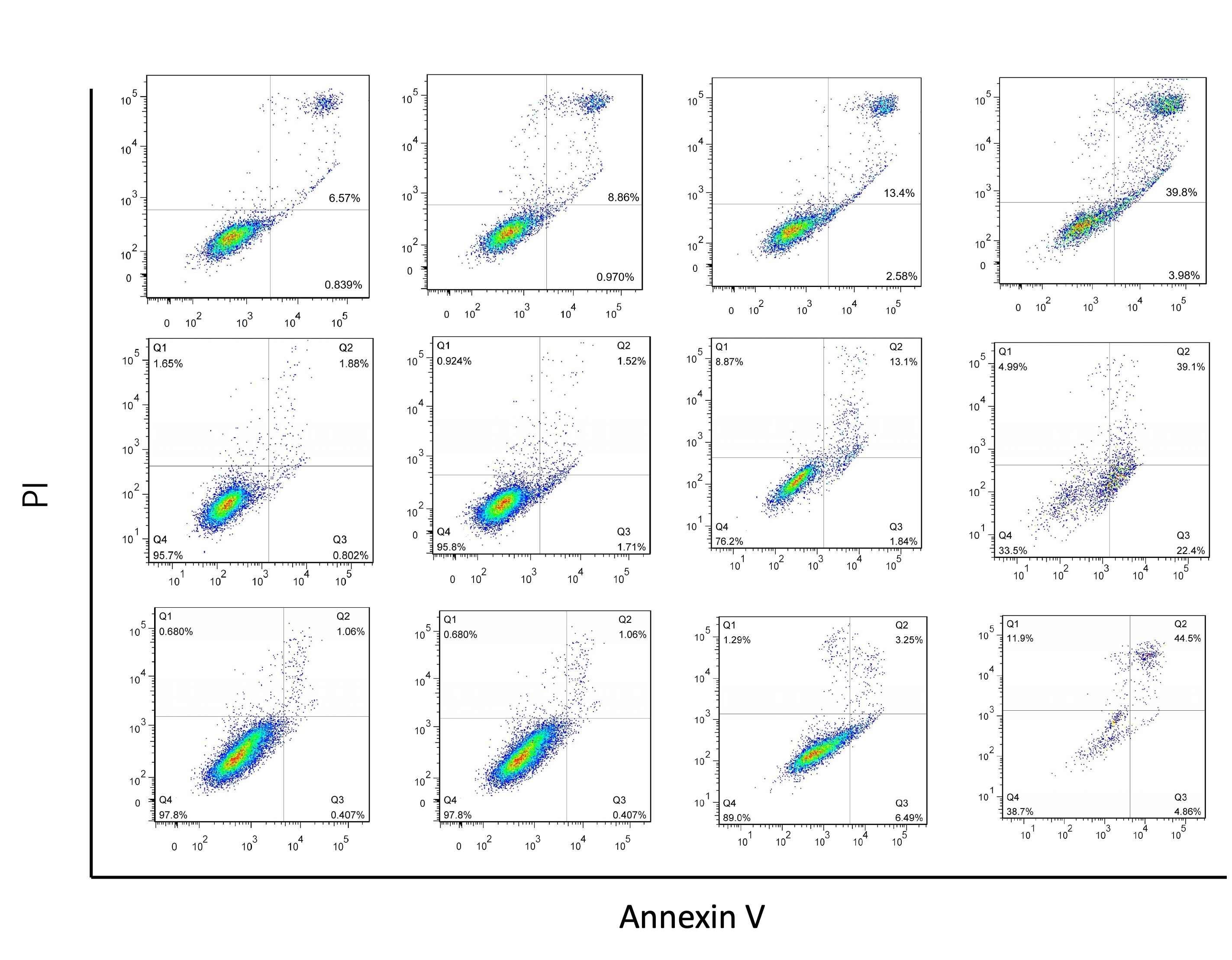

Supplement: Figure S1 [file OncolRes-33-50057-s001.tiff]

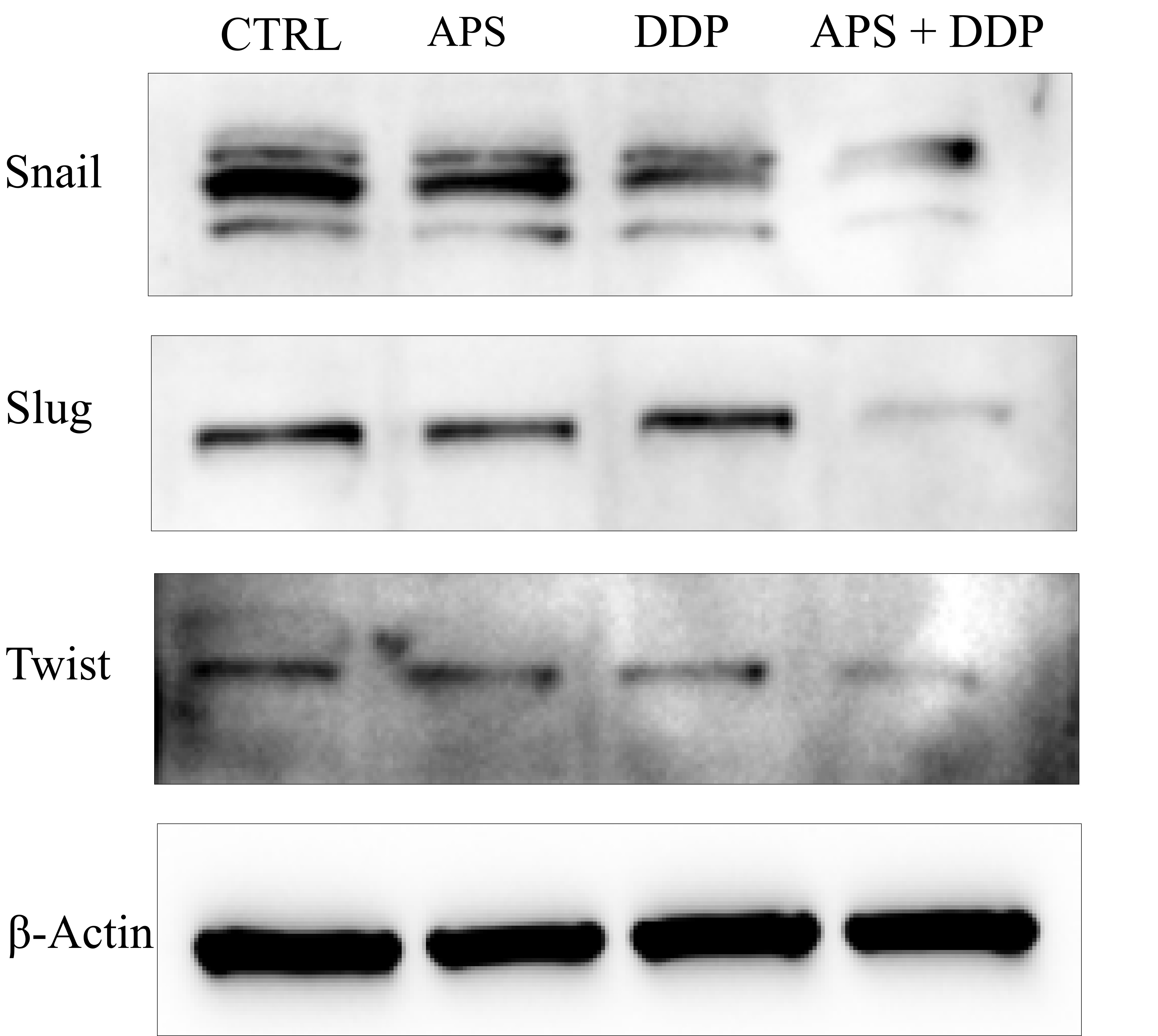

Supplement: Figure S2 [file OncolRes-33-50057-s002.tif]
